# Impact of urbanisation and environmental factors on spatial distribution of COVID-19 cases during the early phase of epidemic in Singapore

**DOI:** 10.1038/s41598-022-12941-8

**Published:** 2022-06-13

**Authors:** Murali Krishna Gurram, Min Xian Wang, Yi-Chen Wang, Junxiong Pang

**Affiliations:** 1 Centre for Infectious Disease Epidemiology and Research, Saw Swee Hock School of Public Health, National University of Singapore, National University Health System, 12 Science Drive 2, Singapore, 117549 Singapore; 2 Department of Geography, National University of Singapore, Block AS2, 1 Arts Link, Singapore, 117570 Singapore

**Keywords:** Diseases, Health care

## Abstract

Geographical weighted regression (GWR) can be used to explore the COVID-19 transmission pattern between cases. This study aimed to explore the influence from environmental and urbanisation factors, and the spatial relationship between epidemiologically-linked, unlinked and imported cases during the early phase of the epidemic in Singapore. Spatial relationships were evaluated with GWR modelling. Community COVID-19 cases with residential location reported from 21st January 2020 till 17th March 2020 were considered for analyses. Temperature, relative humidity, population density and urbanisation are the variables used as exploratory variables for analysis. ArcGIS was used to process the data and perform geospatial analyses. During the early phase of COVID-19 epidemic in Singapore, significant but weak correlation of temperature with COVID-19 incidence (significance 0.5–1.5) was observed in several sub-zones of Singapore. Correlations between humidity and incidence could not be established. Across sub-zones, high residential population density and high levels of urbanisation were associated with COVID-19 incidence. The incidence of COVID-19 case types (linked, unlinked and imported) within sub-zones varied differently, especially those in the western and north-eastern regions of Singapore. Areas with both high residential population density and high levels of urbanisation are potential risk factors for COVID-19 transmission. These findings provide further insights for directing appropriate resources to enhance infection prevention and control strategies to contain COVID-19 transmission.

## Introduction

COVID-19 is a novel viral disease caused by the severe acute respiratory syndrome coronavirus-2 (SARS-CoV-2). COVID-19 is declared a Public Health Emergency of International Concern and a pandemic on 30th January 2020 and 11th March 2020 respectively, by the World Health Organization (WHO). The disease was first reported in Wuhan, Hubei province of China. Bats are believed to be the primary hosts and pangolins as carriers^1^. Even though effective vaccines are available by early 2021 to reduce the risk of COVID-19, implementing social distancing measures and good hygiene practices remain critical to control potential transmission as the pandemic evolves^2–4^.

Prompt hotspot identification and geospatial risk assessment of COVID-19 are critical for strengthening surveillance and public health measures to contain transmission. Recent studies conducted in China have demonstrated the utility of geographic information systems (GIS) for analysing the spatio-temporal trends of COVID-19 epidemic^5–7^. COVID-19 transmission, vitality, stability and infectivity have been directly related with various environmental factors ^8–10^. Climatic factors like temperature, rainfall, humidity, and wind speed could have potential influence on the prevalence and its spread of COVID-19^10–12^. Some attempts have also focused on the impact of urban development and density on the spread and mortality rates of COVID-19^13–15^. This is especially since poor ventilation and drainage systems in buildings facilitated SARS virus transmission during the 2002 SARS outbreak^16^. Nonetheless, mixed results were observed when these evidence were reviewed collectively, for both climatic and human factors^10–15,17^. Salom et al. (2021) observed negative correlations between temperature and humidity with COVID-19 incidence, while positive correlations were reported by Kong et al. (2021)^10,11^. Different states within South Korea presented contrasting correlations between temperature and atmospheric pressure on incidences, while atmospheric pressure was not correlated to COVID-19 incidence in another study of 118 countries^10,14^.

Since the early outbreak of COVID-19, nations across the world have adopted physical distancing as the effective means to combat its spread. Though spatial separation of the people is partly behavioural, it could be greatly influenced by population density^13^. Population density has been identified as one of the key attributing factors for COVID-19 spread in Turkey, Algeria and Italy ^13,18,19^, and an effective predictor of cumulative COVID-19 infection cases in American counties and in the regions of India^20,21^. Nonetheless, the association between population density and incidence have been refuted, with evidence indicating larger influence from the human development index and (percentage or absolute) number of residents living in urban agglomerates on COVID-19 incidence^10^. Regardless, current evidence collectively suggests a potentially more complex interplay between environmental and/or population parameters on COVID-19 incidence, thus requiring alternative approaches to further elucidate the interactions.

The whole premise of this study is that though an epidemic is a biological event, its transmission is driven by various socio-cultural, physical and associated factors^22^. It is worth noting that urban hubs provide the impetus for human development, with high densities of population, concentrated forms of built-up features and related services. However, it is imperative to ensure these development hubs should not become ideal environments to spread infections^23–25^.

Previous studies took a prognostic and curable approach to address the pandemic across broad regions or states. However, this study attempts to unravel the dynamics of COVID-19 spread in the urban environmental context in finer resolutions at neighbourhood level (i.e., subzones, the smallest zonal unit used in urban planning in Singapore) through a spatial analytical approach, which has gone unremarked in discussions. The analysis enhances the current understanding and transmission of the pandemic, and subsequently contributes to implementing better mitigation strategies especially in middle-income countries with tropical climates. In that direction, in relation to environmental and epidemiological characters, the study primarily focused on revealing spatial patterns, relations, geographical spread, dependency and intensity of COVID-19 in the respective sub-zones of Singapore using geospatial modelling and analysis techniques. Specifically, the Geographical Weighted Regression (GWR), which has been reported to be efficient in identifying the spatial variations^26–28^, was employed in this study. The spatial relationships among the sub-zones assessed in this study are (i) between environmental parameters and COVID-19 cases, (ii) between the level of urbanisation and population density and the COVID-19 cases, and (iii) among the three different type of epidemiological cases (linked, unlinked and imported cases).

## Materials and methods

### A. Study area

Singapore, a city-state country, is a prominent business hub in Southeast Asia, home to the regional bases for many global financial and IT institutions. Singapore is also a global transit aviation hub connecting major cities across the globe, with one of the busiest major ports in the world and a high industrial activity. For the above reasons, Singapore attracts thousands of foreigners yearly, mainly from neighbouring countries such as Malaysia, Indonesia, China, Philippines, Vietnam and Thailand, for work and leisure in this city-state country.

Singapore is a densely populated urban centre with a population of around 5.6 million^29^ and a total area of 784 km^2^. The country is located close to the equator(1°17' N, 103°50' E), and experiences typical tropical climatic conditions with abundant rainfall, high and uniform temperatures, and high relative humidity (RH) throughout the year. The climate is primarily characterised by two monsoon seasons that are separated by inter-monsoonal periods. The average temperature in Singapore ranges between 25 and 31 ℃, with an average minimum of 23 ℃ during January and February, and an average maximum of 32 ℃ during April and May^ 30^. RH in Singapore varies widely, from more than 90% in the morning to around 60% in the mid-afternoon on a sunny day, and up to 100% on rainy days. According to the National Environment Agency (NEA), the average RH level in Singapore is 84.2%^30^. The country is divided into a total of 323 administrative sub-zones, which are used as the basis for the location-specific analysis in this study.

### B. Data source and processing

Residential location information (building/street address) of all COVID-19 community cases occurring in Singapore was collected from daily public reports by the Ministry of Health (MOH), Singapore^31^ starting from 23rd January 2020, when the first reported local case, till 17th March 2020 (Supplementary Fig. 1). Only data up to 17th March 2020 were utilised as the required locational information was not released by MOH for cases reported after 17th March 2020. Collected data were geocoded into GIS, based on the residential locations. Cases with only hospital addresses as the residential locations were excluded from analysis. The imported, linked and unlinked cases were subsequently cleaned from syntactical and other errors attributed to locations by employing the address locator function in geocoding which excludes the addresses with syntactical errors while generating the point geometry with corresponding address. Case types were defined based on epidemiological grounds by the Singapore Ministry of Health: (1) linked cases refer to new domestic cases whose potential source of infection belongs to an existing or emerging outbreak cluster (such as, but not limited to, close contacts of previous cases), (2) unlinked cases refer to new domestic cases whose potential source of infection was not linked to any existing or emerging outbreak cluster, and (3) imported cases refer to cases detected from all foreign arrivals or returning travellers^31^. Data on places visited by COVID-19 cases reported publicly from 19th May, when Singapore transited from the lockdown, locally coined as ‘circuit breaker’, to ‘Phase 1’ with eased restrictions, to 31st July 2020 while they were infectious (defined as 14 days prior to case confirmation) were also collected from daily reports by the MOH, to visualise the pattern of places visited by cases in relation to the hotspots detected^31^. Collected data was also converted for mapping through geocoding based on postal codes of places visited.

Data for environmental variables, specifically temperature, RH and rainfall, were obtained from 63 NEA meteorological stations distributed across Singapore^ 30^. For each variable, the monthly averages for respective periods were calculated. The aggregated monthly average temperatures and RH for February and March were considered for evaluation as the majority of the cases (95.8%) included in the analysis were from these two months. The central tendency of the data was checked by calculating variance (σ^2^) to see the variability of values from its mean eventually to identify and exclude the outliers. The derived monthly averages were linked to their corresponding meteorological station locations to perform interpolation. The Inverse Distance Weighted (IDW) method was used in interpolation, to generate continuous raster surfaces showing the spatial variations of the environmental variables. Shepard's method was used with ‘standard’ search neighbourhood option. IDW uses the measured values surrounding the prediction location. The measured values closest to the prediction location have more influence on the predicted value than those farther away. The sub-zones were assigned with alphanumeric codes for easy referential purposes. The generated raster datasets were processed to obtain the average values of individual environmental variables by sub-zones for subsequent GWR analysis. However, due to low variance in rainfall values across sub-zones, a valid outcome cannot be generated due to ‘non-stationarity’ issues hence the parameter was excluded from further analysis with the GWR models.

The total population in each sub-zone in 2018 were obtained from the Department of Statistics, Singapore^32^. The data were then formatted and linked to the GIS layer of sub-zones to compute population density. The level of urbanisation across sub-zones was derived from the percent built-up area of sub-zones derived from built-up land use classes, as proxies. The land use class includes residential areas, business areas, civic-community institutions, commercial areas, educational institutions, hotels, transport hubs, places of worship, utility areas and sports or recreation centres. Sub-zones with more than 50% of built-up areas are classified as urban^33^, and most of the sub-zones fall under the urban category. For the purpose of comparing the relative extent of urbanisation on COVID-19 incidence, we further stratified the extent of sub-zone urbanisation into low level of urbanisation (50–70%), moderate level of urbanisation (70–85%), and high level of urbanisation (> 85%), as defined by the percent built-up area. All the methods were carried out in accordance with relevant guidelines and regulations and the processes related to geospatial data were performed in ArcGIS (version 10.3, ESRI, Redlands CA) unless otherwise specified. Supplementary figure was generated using ArcGIS online version using ESRI base map to generate map outcome.

### C. Analysis

This study aimed to explore the correlation between the spatial heterogeneity of environments (temperature and RH), human populations (density and types of epidemiological cases), and levels of urbanization through geospatial methods. Hotspot analysis was done with a criterion of five or more COVID-19 cases within a geographical range of 200 m distance. This is the smallest possible distance (analytic sphere of influence) at which clustering of incidents was most intense^34^. The GWR, proven to be efficient at identifying the spatial variations^26,35^, was utilised. Typically, the Ordinary Least Squares (OLS) model would be used to analyse the variation in relationship between the incidence rate (IR) of cases within a single locality. However, as the study is evaluating the variation in incidence rate across different sub-zones based on environmental and population parameters, in addition to incidence of case types within each sub-zone, the GWR method was used because the OLS is unable to account for such variations across sub-zones nor assess the aforementioned correlations ^36^. In contrast, GWR is a localized multivariate model allowing parameters of regression estimates to vary locally. Geographically Weighted Poisson Regression (GWPR) is a local form of the Poisson regression with regard to location^37^. Thus, GWR can detect spatial variation of correlations in a model and produce maps for exploration and interpretation of spatial non-stationarity^38^. In this study, the GWR was defined by the spatial relations between neighbouring sub-zones and is parametrically denoted as:

$${\text{y}}_{{{\text{i}} }} = \, \beta_{{{\text{i}}0}} + \Sigma^{{\text{m}}}_{{{\text{j}} - {1}}} \beta_{{{\text{ij}}}} {\text{X}}_{{{\text{ij}}}} + \, \varepsilon^{{\text{i}}} ,{\text{ i }} = { 1},{ 2}...,{\text{ n}}$$

where, y_i_ signifies the incidence of COVID-19 at a specific sub-zone, β_i0_ is the intercept, β_ij_ is the jth regression parameter, X_ij_ is the value of the jth explanatory parameter, and ε^i^ is a random error term. The analysis accounts for characteristics of variable population being evaluated, distance among the sub-zones and spatial distribution. The observations nearer to *i* were given greater weight than those further away. Adaptive kernel was used in combination with bandwidth parameter and nine neighbourhoods were used as the criteria for running the GWR model. GWR explicitly models spatial variation, but it violates the assumption of predictor independence because the same location can be included in different local parameter estimates, which may lead to issues deriving goodness-of-fit statistics. This issue was addressed by extending the standard regression approach by a distance-based weighting by performing local regression analysis of each unit area being evaluated. Significance levels which are the standardized correlation coefficients or ‘z values’ are determined based on the following GWR standardized residual ranges: negatively significant (< −0.5), no significance (−0.5 to 0.5), low significance (0.5 to 1.5), moderate significance (1.5 to 2.5), and highly significant (> 2.5)^38,39^. Significant GWR results indicate varying incidence rate across sub-zones (non-stationary incidence rate/non-stationarity), and also the strength of correlation between the incidence rate (dependent variable) and the factor evaluated (independent variable), if present i.e. GWR ≥ 0.5. A GWR result of low significance indicates low strength in correlation between the incidence rate and evaluated factor, likewise for moderate and high significant GWR results.

The GWR model was used with a basic assumption that study observations were independent of each other. The error estimation is done by Weighted Least Squares (WLS) which gives different weighting to each location and the variance of the error is known. In addition, for the GWR analysis an optimal bandwidth of nine neighbouring subzones was chosen which is sufficient to negate the effect of ‘low variance’. It was also assumed that the structure of the GWR remains constant across the subzones of Singapore, and there are no major local variations in the estimate of parameters being considered. Therefore the model assumes that the error is normally distributed across subzones and the expectation value is around zero and the variance is constant. Comparing GWR result with Student’s two-tailed t-test values determine any significant parameter affecting the dependent variable^37^.

The GWR analysis was performed to assess factors influencing the variation in COVID-19 case incidence across Singapore sub-zones. The factors assessed include: (i) environmental parameters—temperature and RH; (ii) population parameters—residential population density and level of urbanisation; and (iii) case types–linked, unlinked and imported. For the assessment of case types, analysis was performed with three scenarios of combinations of dependent and exploratory variables, namely (a) linked (dependent) versus unlinked (exploratory), (b) imported (dependent) versus linked (exploratory), and (c) imported (dependent) versus unlinked (exploratory).

## Results

### A. Epidemiological characteristics of COVID-19 cases and hotspots distribution during the early epidemic in Singapore

Singapore reported the first imported and local COVID-19 case on 23rd January and 1st February 2020, respectively. A total of 287 cases were reported in Singapore’s residents till 17th March 2020, but only 165 cases had residential information reported and were included in the analysis. The excluded cases either reported the addresses of their hospital (55 cases) or their workplace (67 cases). The mean age of the 165 cases was 47 years old (standard deviation: 15.6 years old, range: 1 to 86 years old), of which 56.5% were males and most were Singapore residents (78.2%, 129 cases). Of the reported cases, 7 cases were reported in January, 67 cases in February, and the remaining 91 cases in March, 2020. Most of the cases were linked cases (75.2%, 124 cases), followed by imported cases (20.6%, 34 cases) and unlinked cases (4.2%, 7 cases).

**Figure 1:**
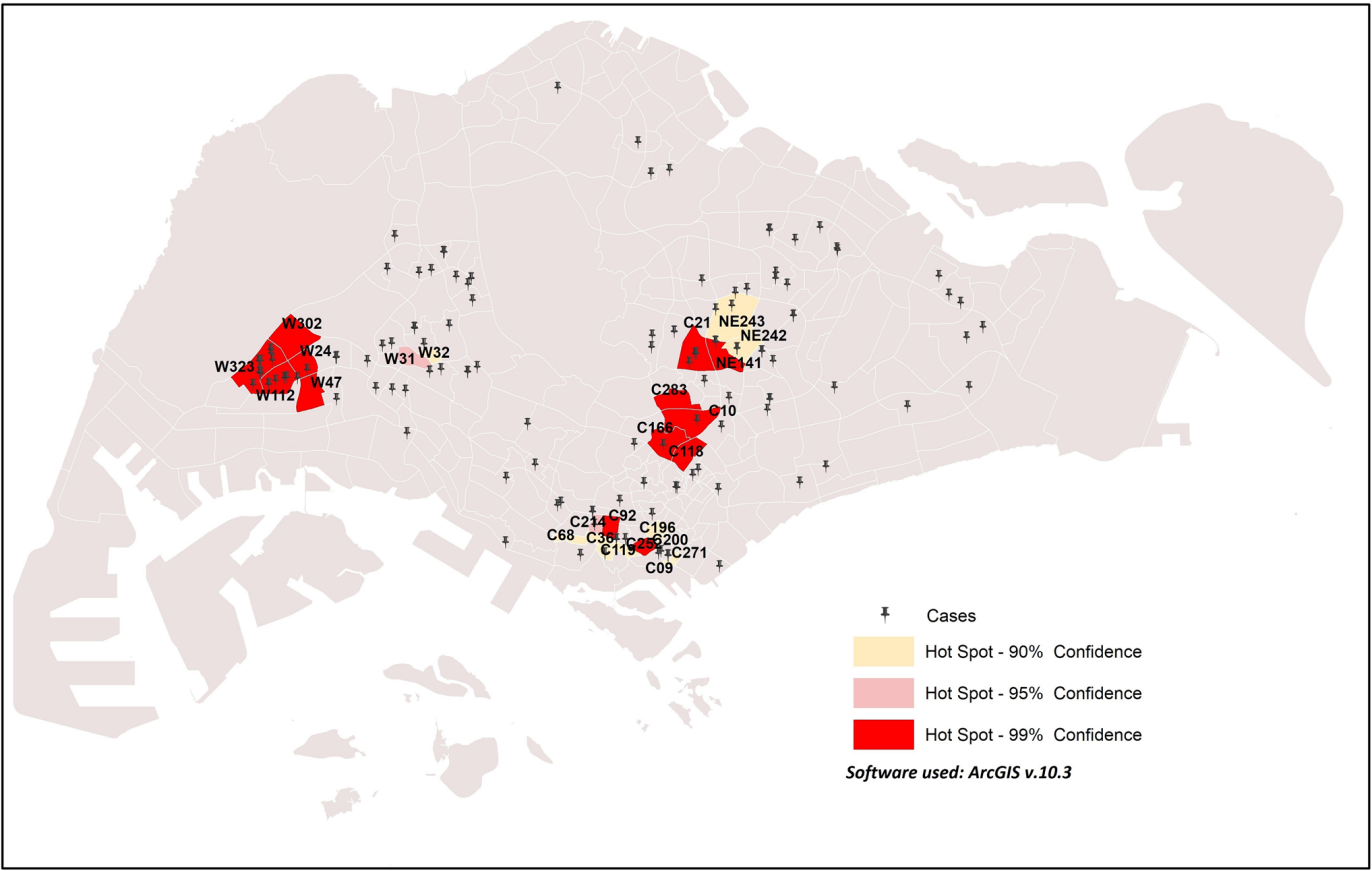
COVID-19 hotspot clustering across Singapore in the early phase of COVID-19 outbreak.

Locations with a relatively high number of cases in comparison to their surroundings are described as hotspots. Hotspot analysis depicts areas with high intensity and concentration of COVID-19 cases during the early phase of the outbreak in Singapore (Fig. 1). Cases were reported in 55 out of the 323 sub-zones in Singapore, and the highest incidence occurred in sub-zone W112 (40 cases). Eight other sub-zones, i.e., C166, C10, C283, C166, C118, C21, NE141 and C92, are also the areas with high incidence of COVID-19.

### B. GWR of COVID-19 with environmental factors

#### i. Temperature

**Figure 2:**
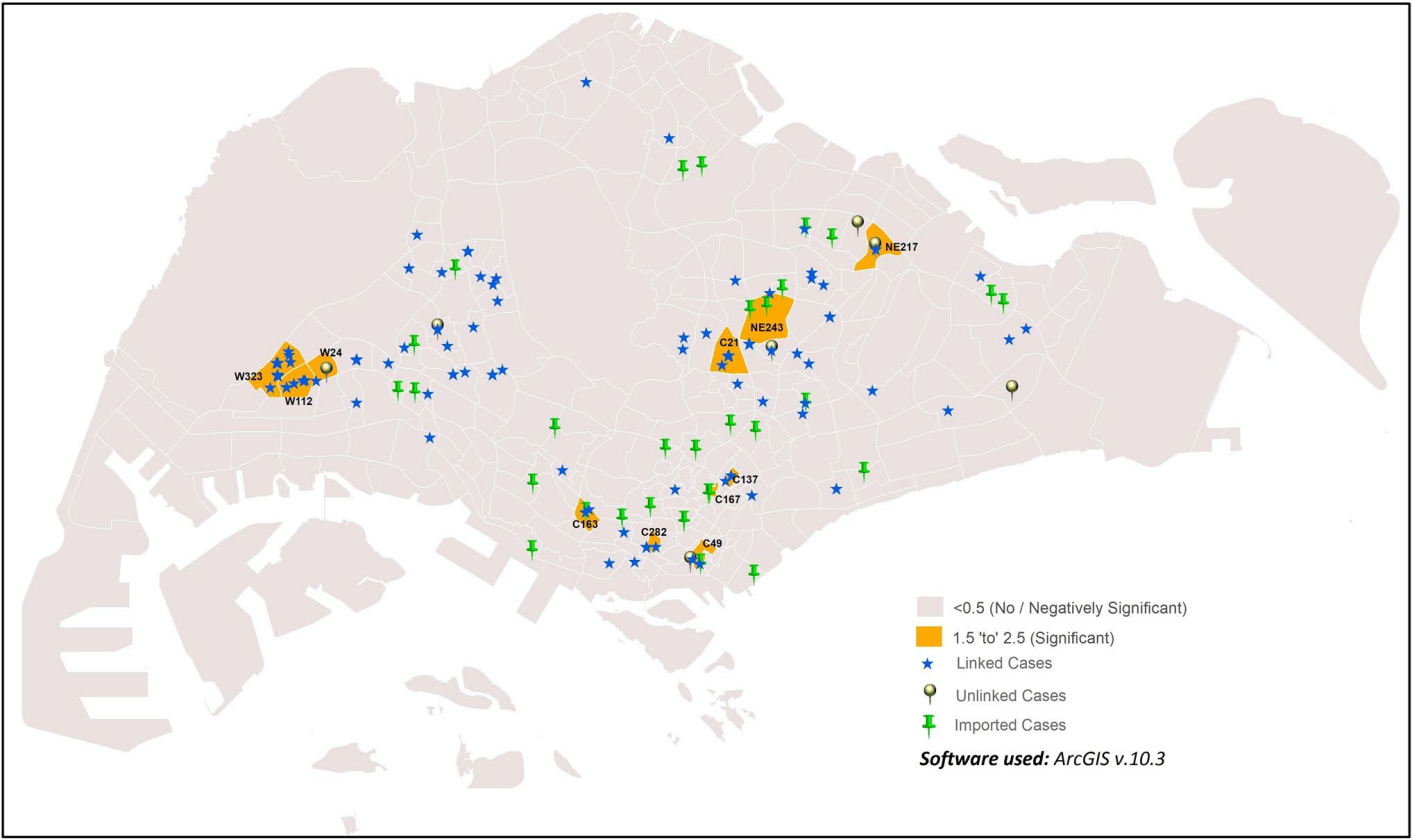
GWR of COVID-19 cases vs. temperature in the early phase of COVID-19 outbreak.

The GWR analysis result showed significant correlation (GWR values = 1.53–1.86) in 11 sub-zones:C21, C49, C137, C163, C167, C282, NE217, NE243, W24, W112 and W323. The GWR results in these sub-zones indicated non-stationarity in incidence rate, and suggested correlation between temperature and COVID-19 incidence (Fig. 2, Supplementary Table 1). However, sub-zones with minimum incidence rates (one or two cases) showed no significance with temperature and hence, lack of correlation with temperature. The remaining sub-zones not reporting any cases showed negative significance due to the absence of COVID-19 incidence.

#### ii. RH

The average RH levels in the sub-zones during February and March 2020 were between 78 and 83%. However, the GWR analysis between case incidence and RH has failed to show any non-stationarity among these sub-zones (Supplementary Table 2). There is no significance found among sub-zones of Singapore, suggesting absence of correlation between RH and cases.

### C. GWR of COVID-19 with population characteristics

#### i. Residential population density

The underlying population density was considered as an exploratory variable influencing the spread and intensity of COVID-19 incidence in each sub-zone. The most densely populated five sub-zones in Singapore are found to be Jurong West Central (46,805 residents/km^2^), Choa Chu Kang (44,879 residents/km^2^), Bukit Merah (44,659residents/km^2^), Bukit Panjang (42,635 residents/km^2^).

**Figure 3:**
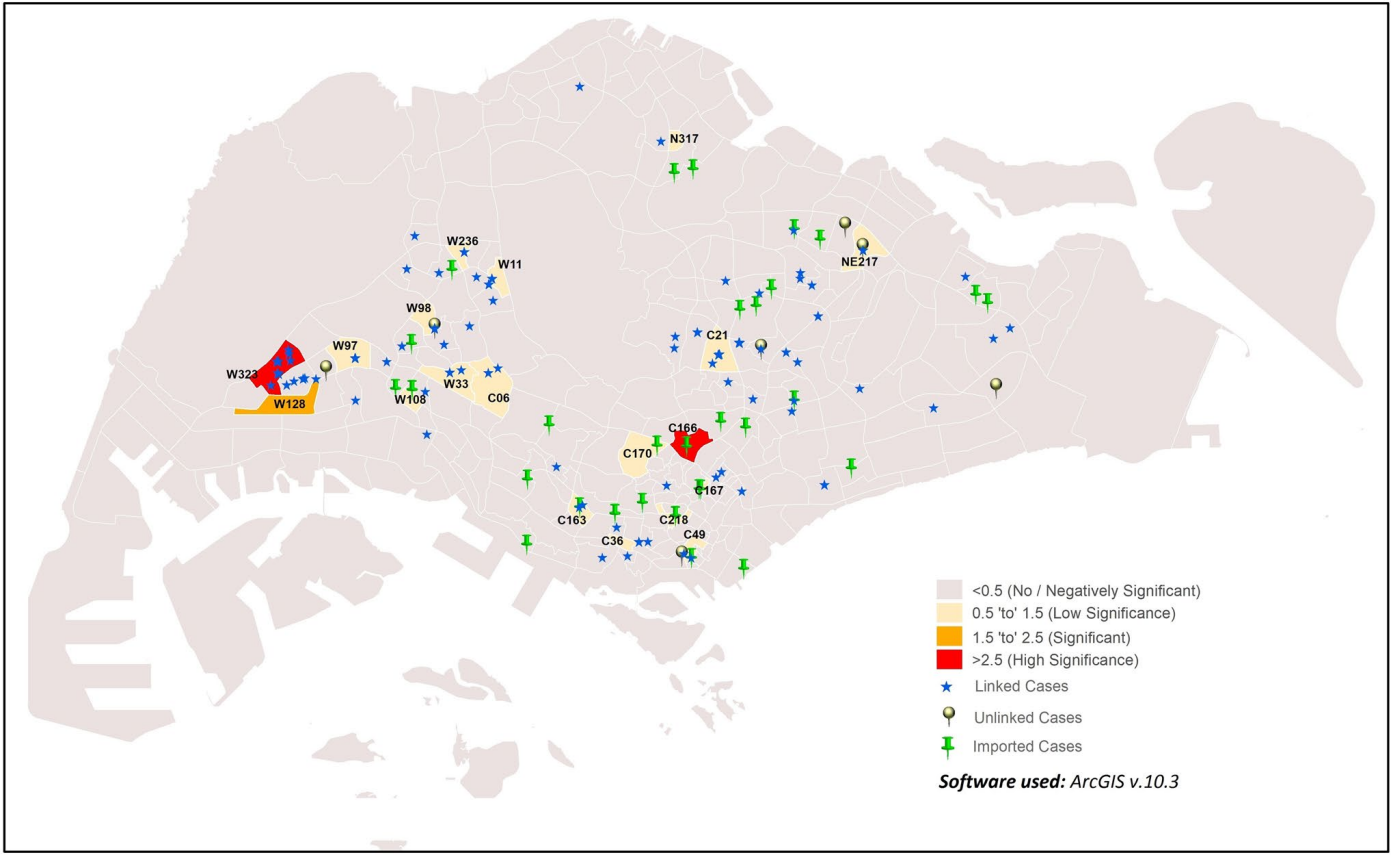
GWR of population density and COVID-19 cases in the early phase of COVID-19 outbreak.

Significant positive correlation was observed between population density and cases in sub-zones located in the western region of Singapore (Fig. 3; Supplementary Table 3). Subzones W323 and W128 had GWR values of 1.96 and 3.89 respectively, suggesting positive significance. High significance was also observed in subzone C166 (GWR value = 2.73). Collectively, the GWR results suggest moderate to high correlation between population density and incidence rate in these sub-zones, suggesting that sub-zone population density is one of the risk factors of potential COVID-19 clustering^40^. In contrast, sub-zones in the remaining regions have not shown much significance.

**Figure 4:**
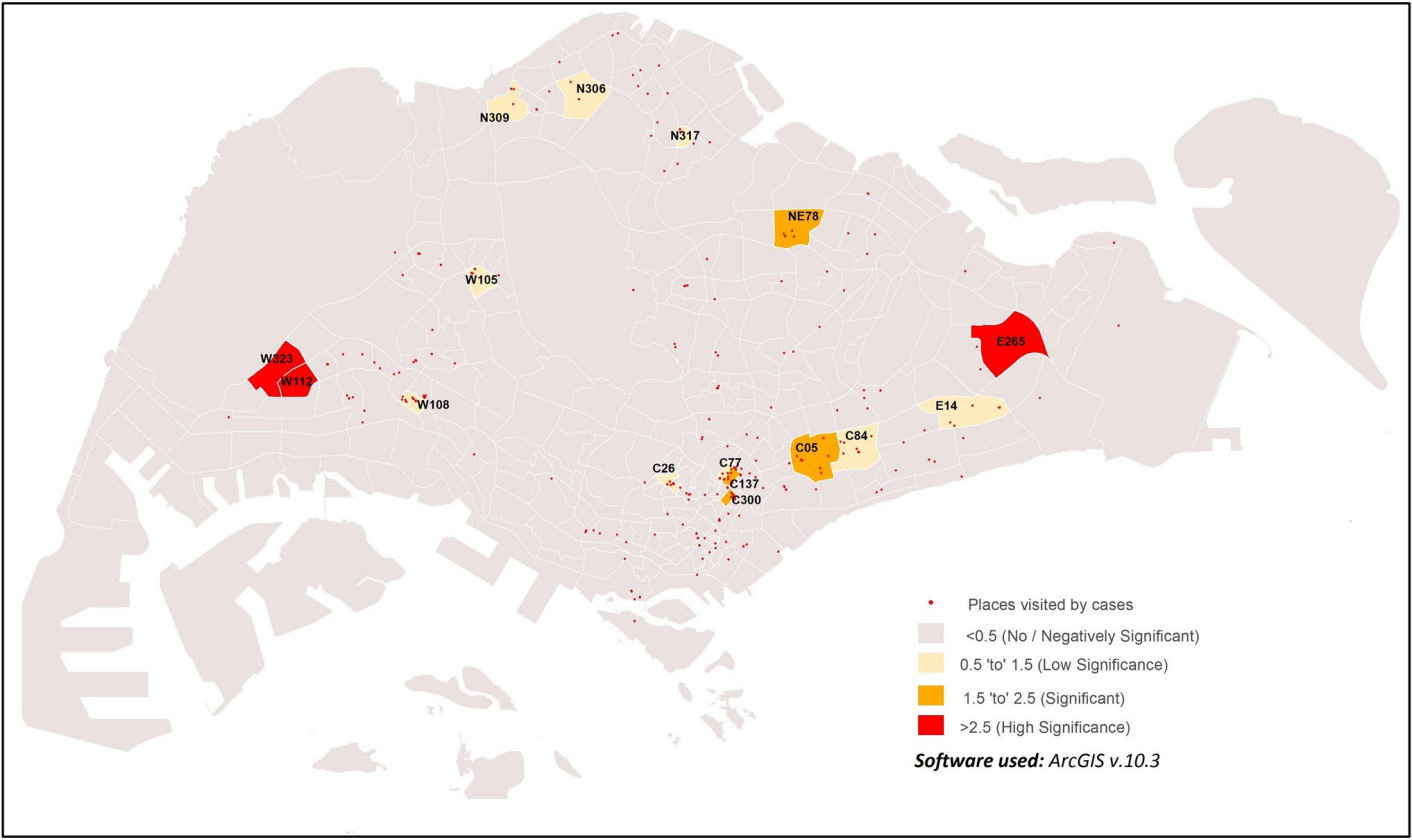
GWR of population density and places visited by COVID-19 cases from 19th May 2020 to 31st July 2020.

The places visited by COVID-19 cases from mid-May to end-July was strongly correlated with population density in three sub-zones, E265, W112 and W323 (GWR values = 3.51 to 5, Fig. 4; Supplementary Table 4). Four sub-zones (C05, C137, C300 and NE78) were found to be moderately correlated with medium significance (GWR values = 1.52–1.62). Weak correlations were observed in nine sub-zones (C26, C77, C84, E14, N306, N309, N317, W105 and W108) with low significance (GWR values = 0.53 to 0.68).

#### ii. Level of urbanisation

The degree of influence and correlation of different levels of urbanisation to COVID-19 incidence were evaluated. Thirteen subzones were found with a high level of urbanisation (> 85%). However, except for W302, E02 and E146, the remaining ten subzones are mostly covered with industries with no cases reported. There were 136 sub-zones at moderate levels of urbanisation (70–85%), and they accounted for 110 COVID-19 cases (45.5%), the highest number of cases among the three levels of urbanisation. Sub-zones in this category of urbanisation include C85, C261, C214, C284, E194, N318, W91, W290 and W323.Ninety-fivesub-zoneswere at low level of urbanisation (50–70%) and they accounted for 81 cases (33.5%). The remaining 79 sub-zones had urban area less than 50%; they accounted for only 44 cases (18.2%), but 20 cases were from C30 sub-zone alone, among which migrant workers living in dormitories made up the majority of the cases.

**Figure 5:**
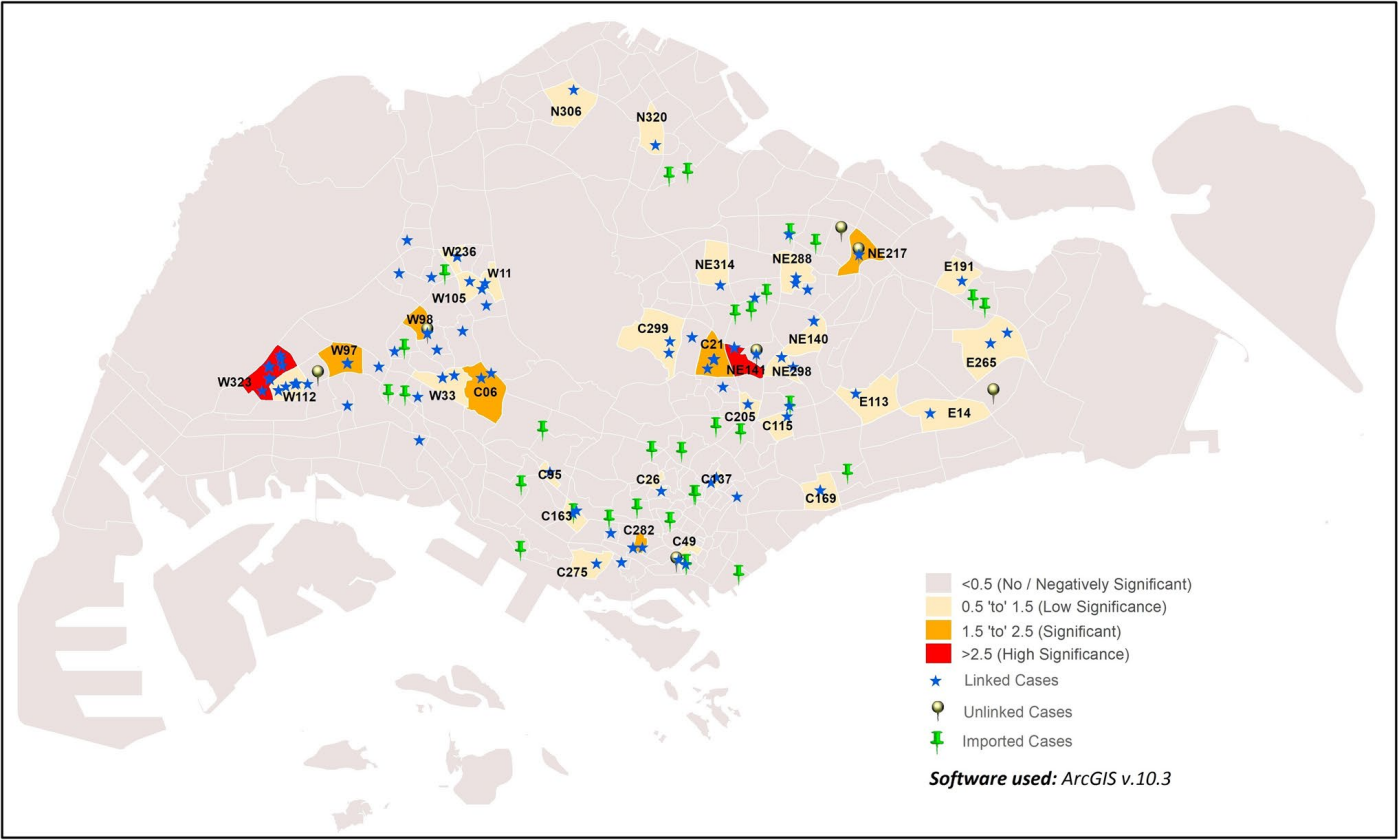
GWR of areas with high levels of urbanisation and COVID-19.

Non-stationarity in COVID-19 incidence was detected in 33 sub-zones, with significance ranging from low to high (Fig. 5, Supplementary Table 5). High correlation between urbanized areas and COVID-19 incidence was suggested in NE141 and W323 sub-zones (GWR values = 2.84 and 10.83, respectively). Medium correlation was suggested in six sub-zones: C06, C21, C282, NE217, W97 and W98 sub-zones (GWR values = 1.56–2.43). Weaker correlation was suggested in 25 other sub-zones. (GWR values = 0.52–1.39). These observations collectively suggest the potential of urbanized areas as a risk factor for COVID-19 cluster formation^41^.

**Figure 6:**
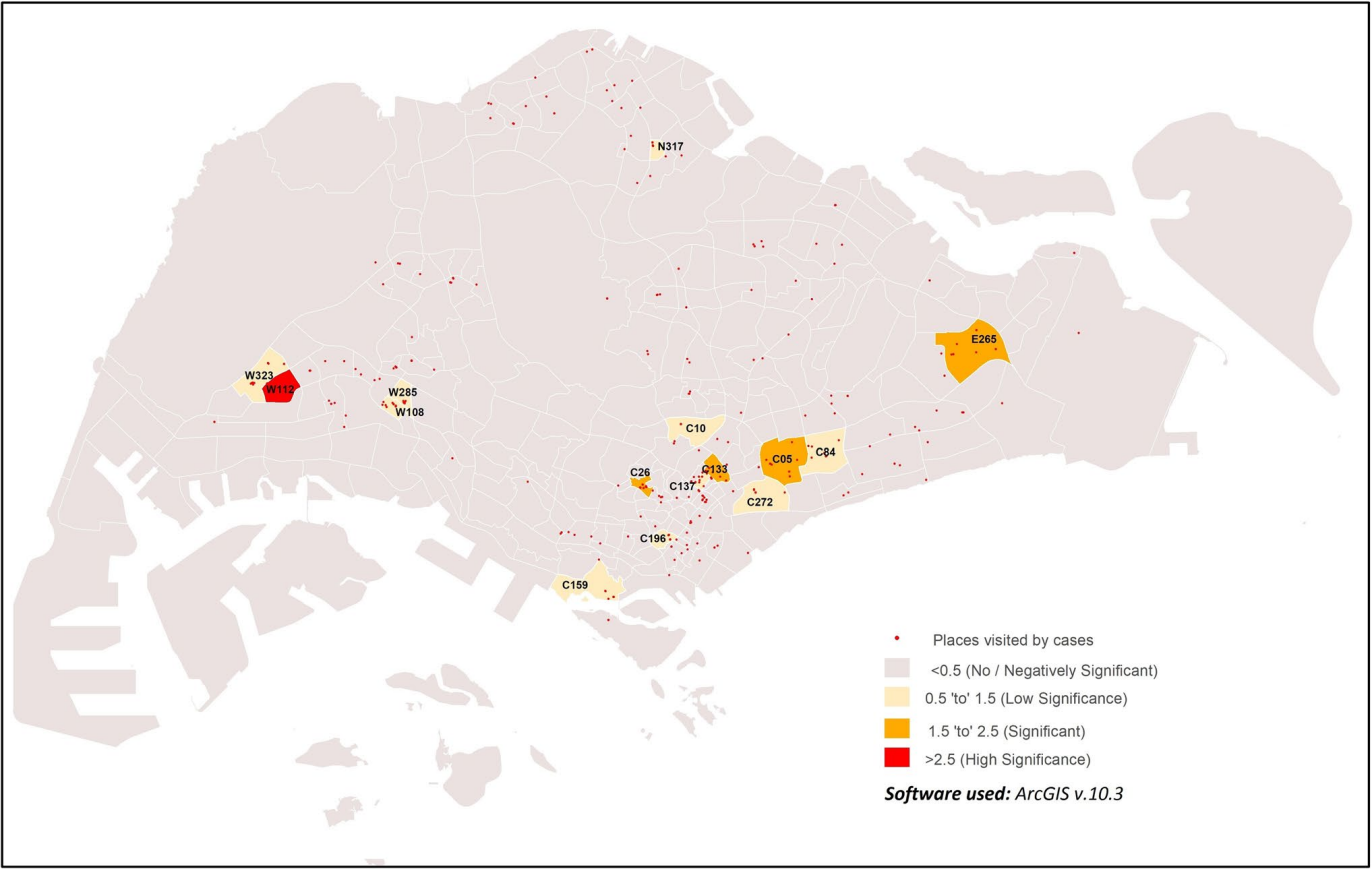
GWR levels of urbanisation and places visited by COVID-19 cases from 19th May 2020 to 31st July 2020.

Spatial analysis of the places visited by COVID-19 cases from mid-May to end-July was suggested to be strongly correlated with the level of urbanisation of sub-zones, in W112 (GWR value = 2.92, Fig. 6; Supplementary Table 6). Similar correlation was observed with moderate strength inC05, C133, C26 and E265 (GWR values = 1.52–1.63). Weak correlations of such nature were also indicated in the following ten sub-zones: C10, C137, C159, C196, C272, C84, N317, W108, W285, W323 (GWR values = 0.5–0.69).

### D. GWR of COVID-19 case type within sub-zone (Linked, Unlinked, Imported)

#### i. Unlinked vs. linked cases

**Figure 7:**
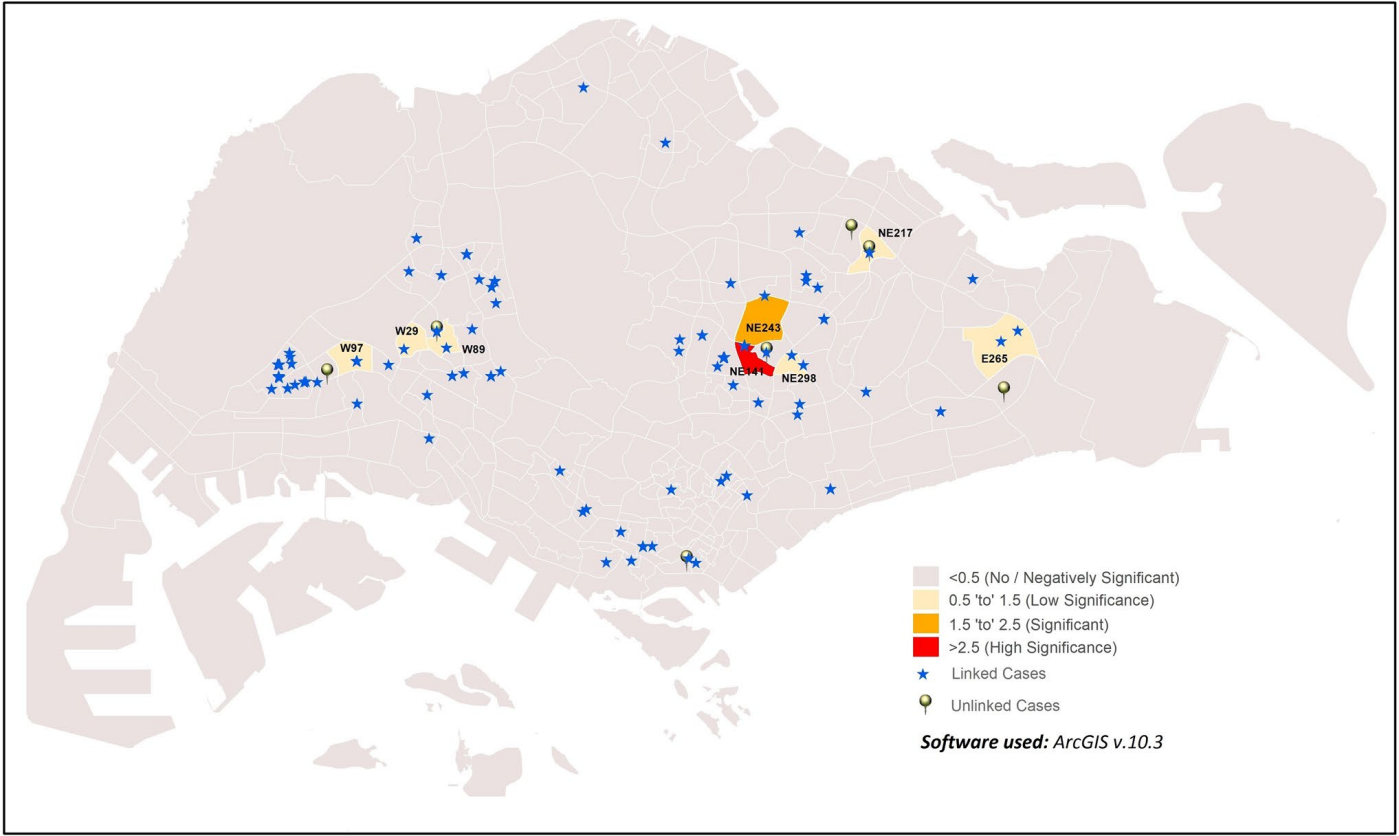
Distribution of sub-zones with correlation of unlinked cases on linked cases.

GWR result of unlinked and linked is shown in Fig. 7. The high significance of linked and unlinked cases in NE141 (GWR value = 5.54), and medium significance in NE243 (GWR value = 1.98) suggests the possibility that unlinked cases can lead to incidence of linked cases in the sub-zone. However, GWR results from E265, NE217, NE298, W29, W89 and W97 sub-zones indicate a relatively lower level of significance (GWR values = 0.51–0.67), suggesting a relatively weaker correlation between the incidence of these two case types within the sub-zones.

#### ii. Imported vs. linked cases

**Figure 8:**
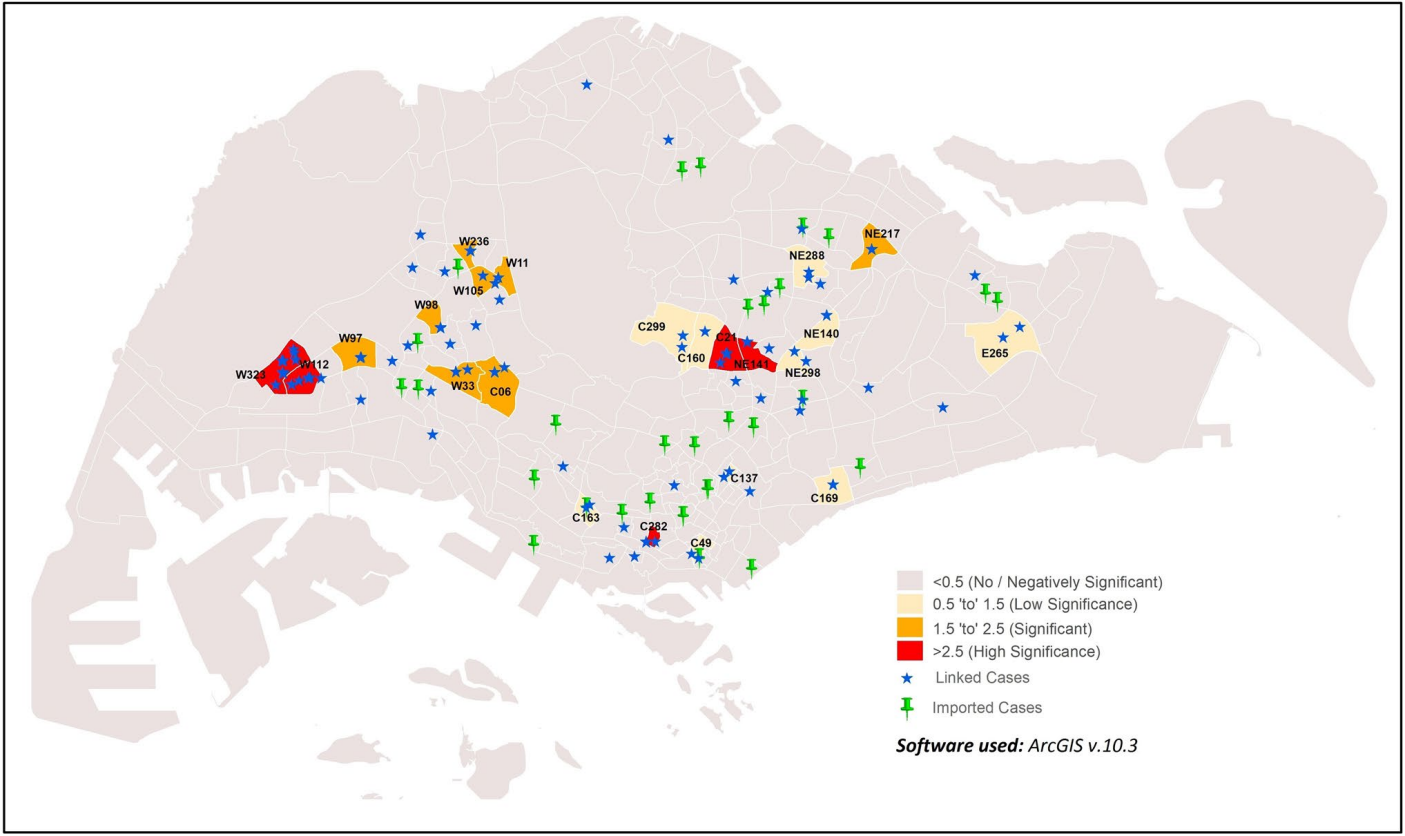
Distribution of sub-zones with correlation of imported and linked cases.

GWR analysis of imported and linked cases showed varied significance across sub-zones (Fig. 8). High significance and correlation was observed in C21, C282, NE141, W112 and W323 sub-zones (GWR values = 3.65–10.06). This suggests plausible association of imported cases giving rise to linked cases in a sub-zone. Moderate correlation (GWR values = 2.05–2.35) was also observed in the following eight sub-zones: C06, NE217, W105, W11, W236, W33, W97 and W98. In contrast, relatively low significance was shown in10 other sub-zones (C137, C160, C163, C169, C299, C49, E265, NE140, NE288 and NE298; GWR values = 0.54–0.97).

#### iii. Imported vs. unlinked cases

**Figure 9:**
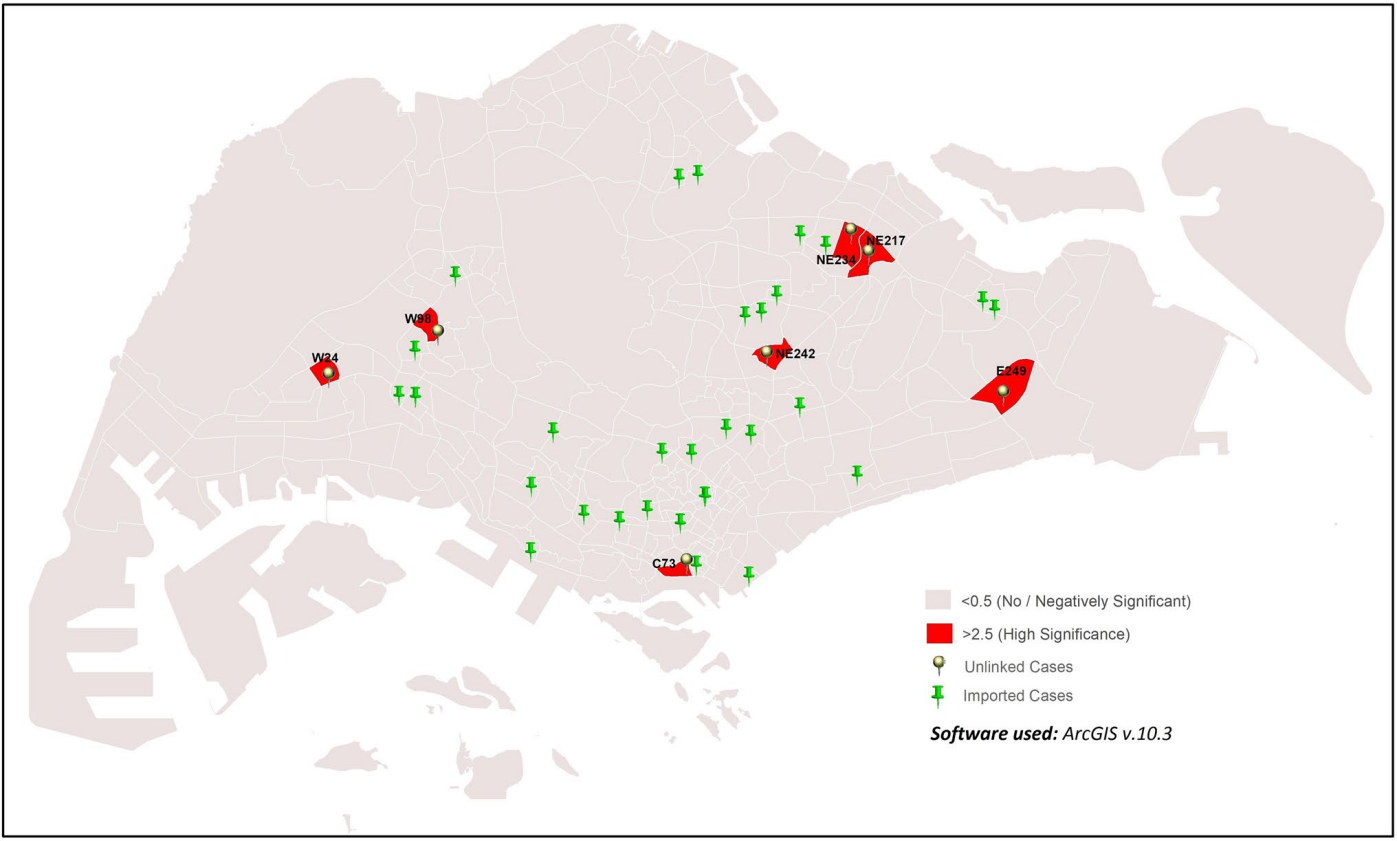
Distribution sub-zones with correlation of imported and unlinked cases.

GWR analysis of imported versus unlinked cases indicated high correlation in C73, E249, NE217, NE234, NE242, W24 and W98 sub-zones (Fig. 9; GWR values = 3.35–3.86). This finding is likely due to close proximity of linked and imported cases in some sub-zones such as in C73, NE217 and NE234. The remaining sub-zones did not show any significance, although 27 other sub-zones reported imported cases.

## Discussion

Geospatial analysis can be a useful tool in supporting outbreak control^42^, especially in identifying disease clusters to direct appropriate resources for implementing control measures^27^. Studies conducted in Wuhan, China and Brazil associated COVID-19 severity with urban related factors^15,43^, while others attempted to investigate the influence of socio-demographic determinants of COVID-19 incidence rates in Oman and Bangladesh using the GWR model^44,45^. Despite these efforts, there is still limited evidence of GWR’s description and application in the ongoing COVID-19 pandemic. The findings of this study added on to the body of evidence in applying geospatial techniques to explore the associations among the COVID-19 hotspots, urbanisation, population density and the climatic factors. Different from prior work, the study further explored potential correlations between incidences of case types within sub-zones. This study also assessed the combined effect of climatic and population factors on COVID-19 incidence within each sub-zone, while previous work generally assessed the sole influence of population or climatic factors^46^. This study supports literature performed in cities with similar population and urbanisation characteristics (e.g. metropolitan, highly urbanised built environment, hyper-dense globalised population) such as London and Wuhan. However, this study also explores a different climatic facet given Singapore’s tropical climate, compared to those cities with temperate climates^28,47^.

### i. Levels of urbanisation and population density as risk factors of COVID-19 hotspots

Identifying hotspots helps to understand the underlying factors responsible for disease transmission, especially for diseases whose transmission are highly correlated with environment and socio-demographic characteristics^27^.

The clustering and hotspot analysis in this study identified sub-zoneW112 as a common hotspot reporting most cases across Singapore during the early phase of the epidemic, and this could be due to the high population density and human activity levels in the sub-zone^40^. As seen from the movement of COVID-19 cases from May to July (Figs. 4 and 6), areas with higher population density and levels of urbanisation are correlated with visits by COVID-19 cases. This is especially so for sub-zone W112 even from May to June 2020, supporting the identification of this sub-zone as a hotspot even from the early pandemic phase in Singapore. A more developed area provides facilities that act as hubs of human congregation and results in relatively higher human activity levels. Thus, it is not startling that 93.8% of COVID-19 cases were reported from sub-zones defined as urban in this study. Associations of increased COVID-19 incidence in districts with high population density or commercial and economic activity spaces were previously observed in Wuhan, China^48,^^49^. In Tehran, Iran, spatial heterogeneity of COVID-19 incidence was observed across the urbanised and highly connected provinces^50^. High population density and level of urbanisation, whether considered singularly or in combination, increases contact rate and social interactions between people, may increase the risk of local transmission in the absence of social distancing practices^51,52^.

Similarly, sub-zones commonly visited by the COVID-19 cases in Singapore had high population density and high levels of urbanisation. These sub-zones in various regions of Singapore include W112 and W323 in the West, C05, C26, C84 and C137 in the South, and E265 in the East.

Clusters reported in March 2020, after 17 March 2020 included a bar in C22 sub-zone, pre-schools in C70 and E14 sub-zones, a private residential compound in C96 sub-zone, and workplaces in C84 and N177 sub-zones. From 3rd week of March 2020 onwards, clusters were increasingly identified in densely populated migrant workers dormitories, starting from dormitories located in NE301 and W286 sub-zones^53^. These clusters were identified in areas with a high level of human congregation and contact between people. This further highlighted high residential population density and high levels of urbanisation are risk factors for disease transmission. In addition, it is worthy to note that the total population reported for each sub-zone excludes the migrant worker population living in dormitories in that sub-zone ^16,54,55^. Thus, the geographical risk in Singapore where such dormitories are congregated, are likely to have been underestimated.

Population movement between urban and rural areas due to urbanisation that brings around work or economic activities, especially via public transport has been identified as a risk factor for disease transmission and cluster formation, regardless within and across cities ^28,47^. In megacities such as London and Wuhan, travelling long distances daily from a hyper-dense residential cluster to another area for work is common. Such great movement daily without any precaution increases the risk of infection while commuting, and the rate of disease transmission at home or workplaces, and through the city. This risk of cluster formation at the destination is further heightened when outbound travel occurs from a region with COVID-19 incidence ^28,47^. Across Singapore, there are sub-zones which are relatively less developed for economic use and more for residential purposes and those where economic activities are concentrated (e.g. the central business district, business parks and industrial parks). However, these sub-zones with different purposes are mostly integrated and well-distributed across Singapore. Thus, there is no obvious segmentation between urban and rural areas in Singapore, excluding Pulau Ubin (an offshore island) and Kampung Lorong Buangkok (which is surrounded by urban residential estates on mainland Singapore). This increases the complexity in studying the effect of population movement due to urbanisation in Singapore. Nonetheless, this study did attempt to study the effect of population movement on COVID-19 incidence through the effect of imported and unlinked cases leading to a cluster of linked cases within a sub-zone.

###  Environmental variables spatially correlated with COVID-19 incidence

GWR result of COVID-19 incidence and average monthly temperatures in February and March shows non-stationarity in incidence, and a weak correlation between these two variables in 11 different sub-zones of Singapore. This suggests that slight differences in average temperatures ranging from 27.5 to 28.5 °C in these sub-zones (C137, C163, C167, C21, C282, C49, NE217, NE243, W112, W24 and W323) were likely more favourable for transmission, compared to that in other sub-zones during February and March (average 27–28 °C). Temporal analysis found a relatively strong positive correlation between average or minimum temperatures, and the total and new COVID-19 cases reported across Singapore during the early phase of the outbreak, between February till end-April^50^. Similar analyses conducted in various countries have also suggested the association between temperature and COVID-19 incidence, although observations may be conflicting^56,57^.The negative effect of temperature on COVID-19 incidence and transmissibility is well-observed globally. Temporal analyses of data from Brazil, China and 166 countries (excluding China) reveal decreased incidence with increased temperature^58^. Across 122 Chinese cities, an approximately inverse linear relationship between temperature and incidence was observed when mean temperature was below 3 °C. However, it is worthy to note that this relationship was negligible above 3 °C and there is no existing evidence supporting the decline in COVID-19 incidence with warmer weather^58^. The observed inverse relationship could also be attributed to decreasing COVID-19 incidence from widespread implementation of rigorous public health interventions, such as city lockdowns and enhanced personal protection measures to control disease transmission, coinciding with increased temperature across China^56,58,59^. In contrast, a study on Wuhan suggests a positive association between diurnal temperature and COVID-19 deaths^59^.

However, the same study also reported decreased COVID-19 deaths when temperature and absolute humidity increased in tandem^37^. Humidity was consistently observed to be negatively associated with COVID-19 incidence in temporal analyses globally^56,58,60,61^. The average RH levels in sub-zones showing correlation with temperature are mostly between 76.5 and 80%, indicating a conducive environment for the virus viability^62^. However, non-stationarity for COVID-19 incidence and RH was not observed, suggesting the lack of any relationship between these two variables in Singapore. Temporal analyses of RH and COVID-19 incidence within Singapore show a weak but positive significant correlation between these two variables, but only in the later outbreak phases after April^63^. This absence of relationship could also be attributed to either severe global or severe local multi-collinearity in RH (explanatory variable) values across sub-zones which are redundant in the GWR model^64^.

Nonetheless, these studies collectively demonstrate the plausible temporal correlation of environmental conditions on COVID-19 transmission. The spatial correlation between higher average temperatures and higher incidence around Tehran was reported by a preprint study in Iran^45^. The potential influence of environmental parameters on the diseases’ morbidity and mortality was suggested to be linked with decreased stability of corona viruses^47,61^ and strengthened host immunity due to associated habits and activities in warmer and humid weather^62,64^. The infectivity of corona viruses was shown to decrease with temperature; a 4-log reduction observed when thermal disinfection at 60 °C for 30 min and only requiring 1 min when 80 °C was used^56^. However, infectivity of the middle east respiratory syndrome coronavirus was shown to be stable at room temperature of 25 °C for at least 2 h^61^. Another study on the survival rates of severe acute respiratory syndrome coronavirus suggests that the virus becomes inactive at temperatures < 20 °C and > 40 °C and RH levels that are < 20% or > 80%^65^. In the context of the current pandemic, a preprint suggested that the SARS-CoV-2 is stable at 37 °C for at least 24 h and is only activated after heating at 56 °C for 30 min^54^. Thus, unless extremely high ambient temperatures are present (such as in the desert), the spatial correlation of temperature with COVID-19 incidence may not be as easily observed.

The influence of climatic factors on COVID-19 prevalence could have far-reaching consequences for public health in terms of policies and formulating mitigation strategies ^55,66^.Contaminated droplets can travel relatively further(up to 6 m in 5 s) under moderate to low temperatures below 20 °C, compared to higher temperatures above 30 °C. In higher temperatures, droplets evaporate faster and hence could travel only a relatively shorter distance^67^. RH affects aerosol particle size. Dry air keeps aerosols smaller, allowing infectious aerosols to stay airborne for longer periods. Likewise, larger particle size when the air is humid, results in shorter time airborne as aerosols become larger and heavier and they quickly fall to the surface. Weather has also been shown to greatly influence humans’ behaviour, activity patterns and preference for activities through the day^68–70^.In particular, extreme weather generally decreases an individual’s level of mobility, especially when public transport systems are less developed, and their preference to stay indoors for longer durations^68^. On a day with very cold (−5 °C to 5 °C) and non-windy (wind speed < 2 km/h) weather, a studied population in Tokyo was found to prefer spending longer durations indoors, especially at food and beverage outlets and to a lesser extent, retail spaces^68^. The clustering of such facilities is more likely with increasing extents of urbanisation. This further escalates disease transmission risk and possibly influenced the correlation of COVID-19 incidence with high levels of urbanisation, as observed in this study. To avert potential harms of extreme heat on health, even WHO advised the public to stay indoors or in a cool or air conditioned area for extended durations during a heat wave^71^. While the perception of heat varies across climates, studied sample of 1508 Singapore residents generally felt that temperatures between 21.6 and 31.6 °C (mean temperature 26.6 °C) were tolerable before they felt uncomfortable from the cold or heat. However, a lower temperature of 24.2 °C was still generally preferred, even by those acclimatised to the heat, while those not acclimatised preferred an even lower temperature of 18 °C ^72^. Hence, the average temperatures of 25 °C to 31 °C through the year in Singapore and the observed average 27 °C to 28 °C in February and March 2020, although tolerable and not considered a heat wave, was possibly still too hot for Singapore residents generally. This increases the potential of Singapore residents possessing habits or routines associated with avoiding the heat, such as staying indoors or in air conditioned public places for extended durations during the day. Such habits to avoid the heat could increase transmission risk, although warmer weather is supposedly associated with decreased COVID-19 incidence^63^. The increased risk is attributed to relatively poorer ventilation and breathing the same air for a long time indoors^73^.Thus, activity patterns arising from warm temperatures beyond comfortable thresholds could be similar to that observed in relatively cold weather (such as in winter). The similar activity patterns in warm or cold weather, with increased transmission risk due to prolonged periods spent indoors, could therefore contribute to higher incidence even during warmer temperatures.

###  Unlinked case incidence spatially correlated with imported and linked cases

The GWR results showed a distinctive spatial heterogeneity of COVID-19 incidence within sub-zones (Figs. Figs. 7, 8, 9), indicating non-stationarity among the linked, unlinked, and imported cases. To a certain extent, the influence of temperature and population characteristics encouraging disease transmission may explain the associations observed between the case types. Some sub-zones showing associations between case types have shown correlations of COVID-19 incidence with at least one of the following parameters: temperature, residential population density and high level of urbanisation in sub-zone, or are in close proximity to sub-zones showing correlation to the aforementioned parameters. These sub-zones include C21, C282, NE78 (next to NE62), NE141, NE217, W97 (in close proximity to W323) and W112. The parameters promoting disease transmission could have potentially amplified the associations between case types in these sub-zones. Nonetheless, the detection of associations between case types even in sub-zones without correlation to these parameters suggests the limited role of environmental and population parameters towards disease transmission within sub-zones.

Compared to environmental and population parameters, the movement of people within their neighbourhood might have assisted disease spread to a greater extent, leading to the observed associations between case types. The presence of imported cases giving rise to linked or unlinked cases within the same sub-zone might be explained by the ability of close contacts of the imported cases, such as family members residing in the same household, or even the case themselves to move around freely around Singapore prior to detection who may lead to pre-symptomatic transmission^74^. Retrospective studies of 3,384 close contacts of confirmed cases in Guangzhou and Shenzhen, China found a mean secondary attack rate (SAR) of 11.2% to 17.1%^75^. The SAR is especially high when contacts were defined on the basis of residential address (mean SAR 17.1%, 95% confidence interval 13.3–21.8%)^72^.In addition, a seroepidemiological study which traced about 2,500 household close contacts of confirmed COVID-19 cases in Singapore found approximately 23% infected contacts to be asymptomatic^76^. This potentially increases the risk of transmission in the residential area. While asymptomatic transmission of the SARS-CoV-2 is still debatable and reported from China at the time of writing^77^, presymptomatic transmission have been reported from Germany, and even also hypothesised as the most likely driver of secondary cases in seven case clusters in Singapore between 23 January to 16 March 2020^74,77^. Likewise, the movement of unlinked cases while presymptomatic within and across sub-zones might lead to the formation of case clusters (i.e. linked cases), as observed in the seven case clusters originating from an unlinked case in Singapore^74^. Furthermore, the likelihood is strengthened when there were instances of residents breaching their ‘leave of absence’ and stay-home notices in Singapore^78–80^. While the residents in these reported instances were shown to be free from COVID-19 eventually, their potential failure to comply with the quarantine measures would have posed a great risk to their local community and potentially result in cluster formation should they turn out to be presymptomatic cases.

###  Limitations

The study was carried out using residential or places visited of COVID-19 cases, which may not fully represent the actual site of disease transmission in Singapore. However, it does represent one of the potential geographical areas for risk of exposure. The sample size was limited as reported by MOH for public use. Activity spaces need to be considered in the current sample for a more comprehensive analysis of population density and urbanisation on the incidence. Limited data were available for validation beyond 17th March 2020 due to the ‘circuit breaker’ implemented in Singapore, which restricted the movement of the community. The places visited of these COVID-19 cases could potentially be subjected to recall bias and it did not account for the frequency of the cases visiting the place. Due to relatively smaller size, the neighbourhoods do not show much variation in the values of factors being evaluated e.g. temperature, rainfall and RH. This resulted in ‘non-stationarity’ of sub-zones, which is the result of low or no variation in the values of exploratory variables. Thus, many sub-zones did not have a significant outcome, despite the presence of COVID-19 cases. Air pollution mighthave influenced an individual’s likelihood of dying from COVID-19. However, this study did not consider the role of individual air pollutants because individual-level COVID-19 data is not readily available. Nevertheless, this study attempted to study the influence of pollution indirectly using the Pollutant’s Standard Index (PSI) values of the subzones. Nonetheless, low variance in 24-h average PSI value range (i.e. 34–57) across the subzones GWR resulted in non-stationarity and did not produce any outcome.

Lastly, the model presented in this manuscript is unable to derive causality and is predictive in nature. Thus, all relationships assessed and referred to in this manuscript are only associations and not causal relationships between the variables and outcome. These associations may also be confounded by residual causal or attributing factors, further limiting the predictive ability of the presented model.

## Conclusions

High residential population density and high level of urbanisation are associated with high risk of COVID-19 incidence. This study may provide valuable insights for outbreak management. Outbreak management through increased active surveillance and outbreak prevention efforts, including wastewater testing and vaccination, in areas with high population density and urbanisation.

## Supplementary Information

Supplementary Table S1.

Supplementary Table S2.

Supplementary Table S3.

Supplementary Table S4.

Supplementary Table S5.

Supplementary Table S6.

Supplementary Figure S1.

Supplementary Legends.
