# Supplementary figures and images for: Impact of urbanisation and environmental factors on spatial distribution of COVID-19 cases during the early phase of epidemic in Singapore
Source: Sci Rep. 2022 Jun 13;12:9758. doi: 10.1038/s41598-022-12941-8 (PMC9191550; doi:10.1038/s41598-022-12941-8)

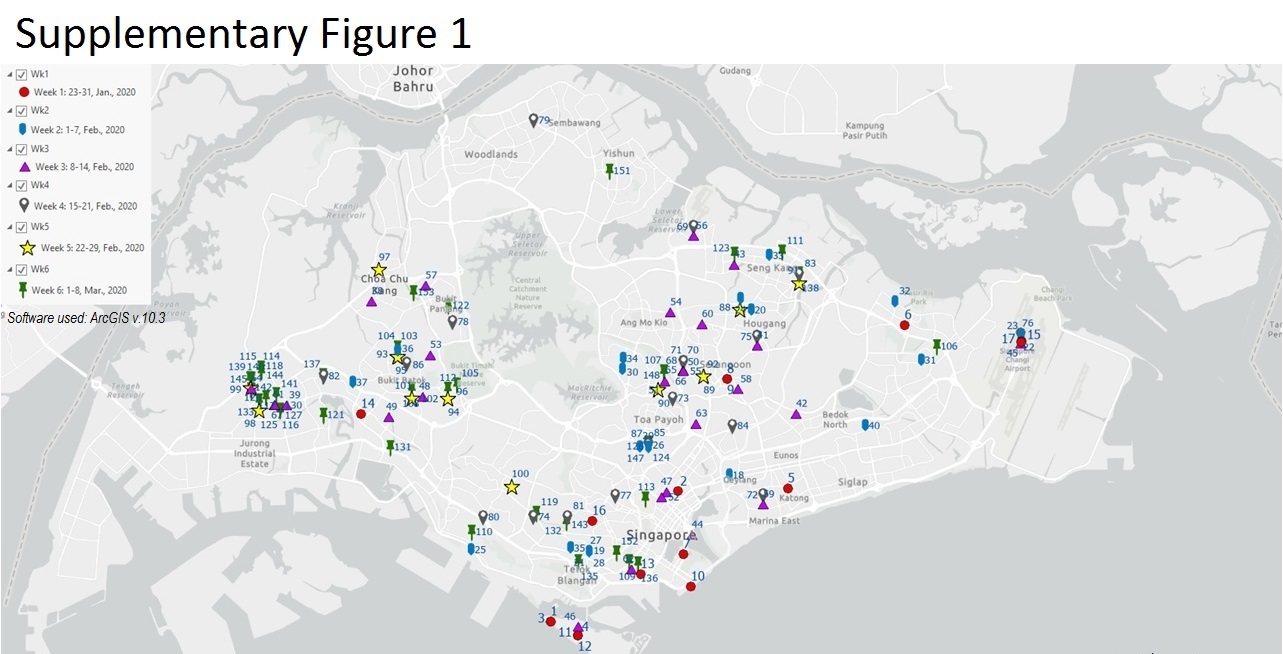

Supplement: Supplementary file 7 — Supplementary Figure S1. [file 41598_2022_12941_MOESM7_ESM.jpg]
