# Supplementary material for: Impact of urbanisation and environmental factors on spatial distribution of COVID-19 cases during the early phase of epidemic in Singapore
Source: Sci Rep. 2022 Jun 13;12:9758. doi: 10.1038/s41598-022-12941-8 (PMC9191550; doi:10.1038/s41598-022-12941-8)
Supplement: Supplementary file 8 — Supplementary Legends. [file 41598_2022_12941_MOESM8_ESM.docx]

# SUPPLEMENTARY DATA

1. Supplementary Table 1 (.xls).
   1. Title: GWR of COVID-19 cases vs. temperature in the early phase of COVID-19 outbreak for all sub-zones.
   2. Description: Table of GWR values (significance of correlation) for temperature against COVID-19 incidence in all sub-zones
2. Supplementary Table 2 (.xls).
   1. Title: GWR of COVID-19 cases vs. relative humidity in the early phase of COVID-19 outbreak for all sub-zones.
   2. Description: Table of GWR values (significance of correlation) for relative humidity against COVID-19 incidence in all sub-zones
3. Supplementary Table 3 (.xls).
   1. Title: GWR of COVID-19 cases vs. population density in the early phase of COVID-19 outbreak for all sub-zones.
   2. Description: Table of GWR values (significance of correlation) for population density against COVID-19 incidence in all sub-zones
4. Supplementary Table 4 (.xls).
   1. Title: GWR of population density and places visited by COVID-19casesfrom 19^th^ May 2020 to 31^st^ July 2020, for all sub-zones.
   2. Description: Table of GWR values (significance of correlation) for population density against places visited by COVID-19 cases from May to July in all sub-zones
5. Supplementary Table 5 (.xls).
   1. Title: GWR of COVID-19 cases vs. level of urbanisation in the early phase of COVID-19 outbreak for all sub-zones.
   2. Description: Table of GWR values (significance of correlation) for level of urbanisation against COVID-19 incidence in all sub-zones
6. Supplementary Table 6 (.xls).
   1. Title: GWR of level of urbanisation and places visited by COVID-19casesfrom 19^th^ May 2020 to 31^st^ July 2020, for all sub-zones.
   2. Description: Table of GWR values (significance of correlation) for level of urbanisation against places visited by COVID-19 cases from May to July in all sub-zones.
7. Supplementary Figure 1 (.jpg).

Title: Map illustrating space-time heterogeneity and weekly progression of COVID-19 incidents in the subzones of Singapore.
